# Effect of a Brain–Computer Interface Based on Pedaling Motor Imagery on Cortical Excitability and Connectivity

**DOI:** 10.3390/s21062020

**Published:** 2021-03-12

**Authors:** Vivianne Flávia Cardoso, Denis Delisle-Rodriguez, Maria Alejandra Romero-Laiseca, Flávia A. Loterio, Dharmendra Gurve, Alan Floriano, Carlos Valadão, Leticia Silva, Sridhar Krishnan, Anselmo Frizera-Neto, Teodiano Freire Bastos-Filho

**Affiliations:** 1 Postgraduate Program in Biotechnology, Federal University of Espirito Santo (UFES), 29075-910 Vitoria, Brazil; loteriofa.ufes@gmail.com (F.A.L.); teodiano.bastos@ufes.br (T.F.B.-F.); 2 Postgraduate Program in Electrical Engineering, Federal University of Espirito Santo (UFES), 29075-910 Vitoria, Brazil; delisle05@gmail.com (D.D.-R.); alejandralaiseca@gmail.com (M.A.R.-L.); carlostvaladao@gmail.com (C.V.); araujos.leticia@gmail.com (L.S.); anselmo@ele.ufes.br (A.F.-N.); 3 Department of Electrical, Computer, and Biomedical Engineering, Ryerson University, Toronto, ON M5B 2K3, Canada; dgurve@ryerson.ca (D.G.); krishnan@ryerson.ca (S.K.); 4 Federal Institute of Espırito Santo (IFES), 29932-540 São Mateus, Brazil; alanspfloriano@gmail.com

**Keywords:** brain–computer interface, brain connectivity, lower limb rehabilitation, motor sensory rhythms, pedaling

## Abstract

Recently, studies on cycling-based brain–computer interfaces (BCIs) have been standing out due to their potential for lower-limb recovery. In this scenario, the behaviors of the sensory motor rhythms and the brain connectivity present themselves as sources of information that can contribute to interpreting the cortical effect of these technologies. This study aims to analyze how sensory motor rhythms and cortical connectivity behave when volunteers command reactive motor imagery (MI) BCI that provides passive pedaling feedback. We studied 8 healthy subjects who performed pedaling MI to command an electroencephalography (EEG)-based BCI with a motorized pedal to receive passive movements as feedback. The EEG data were analyzed under the following four conditions: resting, MI calibration, MI online, and receiving passive pedaling (on-line phase). Most subjects produced, over the foot area, significant event-related desynchronization (ERD) patterns around Cz when performing MI and receiving passive pedaling. The sharpest decrease was found for the low beta band. The connectivity results revealed an exchange of information between the supplementary motor area (SMA) and parietal regions during MI and passive pedaling. Our findings point to the primary motor cortex activation for most participants and the connectivity between SMA and parietal regions during pedaling MI and passive pedaling.

## 1. Introduction

Currently, there is a growing interest in extending research on brain–computer interfaces (BCIs) to a variety of applications, such as to induce neuroplasticity and neural functional restoration. BCIs have received special attention due to their demonstrated potential to treat or assist people suffering neural diseases or neurological disorders [1,2,3]. Several studies have proposed BCI technology as a promising approach to generate activity on the primary motor cortex, offering an alternative way for participants to actively practice the intention of moving their upper and/or lower limbs [4,5,6,7]. As a result, the final device or/and application provides multisensory feedback, enhancing brain rhythm modulation [2]. Thus, BCIs can couple intention with action, where the electroencephalography (EEG) techniques have been widely used in several studies related to lower limb movements [4,5,8,9,10]. This interest of researchers in developing BCIs for the recovery of lower limbs arises from the fact that gait recovery is one of the main objectives of affected subjects since locomotion has a strong impact on quality of life [11]. For this purpose, the use of BCIs based on robotic devices for offering feedback has stood out, since robotic therapy minimizes assistance and encourages the maximum effort of subjects [12,13,14], in addition to allowing a high number of repetitions with accurate movements. Specifically, motorized pedals for pedaling exercises have aroused interest due to their low cost and portability.

Nowadays, technologies with these characteristics are becoming more attractive for motor rehabilitation at home, thus respecting the social distancing [15,16] that is required at this time of the COVID-19 pandemic, which presents a high risk to the elderly and can cause severe neural diseases [17,18,19,20]. In addition, pedaling movements have demonstrated positive results in the functional recovery of lower limbs [21,22,23,24] for a wide range of motor disabilities, presenting the advantage of being safe and accessible [22,25] for post-stroke, Parkinson’s disease, and/or spinal cord injury patients [26,27,28]. Consequently, the interest in EEG-based BCIs for providing pedaling movements is increasing. In fact, previous studies have shown the involvement of the primary motor cortex when subjects perform real (active or passive) or imagery tasks of pedaling movements [29,30,31,32].

On the other hand, studies about brain connectivity have analyzed the dynamic behavior of cortical activity and the roles of different regions during real movements and MI [33,34,35]. However, studies that used EEG-based BCIs for lower limb rehabilitation by performing MI tasks did not report, to the best of our knowledge, how brain regions connect amongst themselves. The cortical connectivity analysis describes the interactions between brain locations through patterns that represent the dynamics of information flow [34,36,37]. Thus, this information may contribute to developing more effective therapy with BCIs by obtaining a classification model based on connectivity from various subjects. In fact, a subject independent BCI based on brain region connectivity was recently proposed for emotion recognition, achieving promising results [38].

Our study aims to analyze the sensory motor rhythm behavior and cortical connectivity through EEG when a BCI is activated by pedaling motor imagery, offering passive pedaling as real-time feedback. Our findings may contribute to future research aiming to develop more robust BCIs and, consequently, therapies, as well as to advances in subject independent BCIs to facilitate their use in clinical environments.

This paper is divided into four sections. Section 2 describes the BCI used in our study. Subsequently, the participants’ demographic data, the inclusion and exclusion criteria, the experimental protocol, and the methodology for data analysis are also presented in this section. In sequence, the results and discussion of how our BCI affected the users’ cortical excitability and brain connectivity are described in Section 3. Finally, the conclusions of our study are presented in Section 4.

## 2. Materials and Methods

### 2.1. Brain—Computer Interface

**Figure 1:**
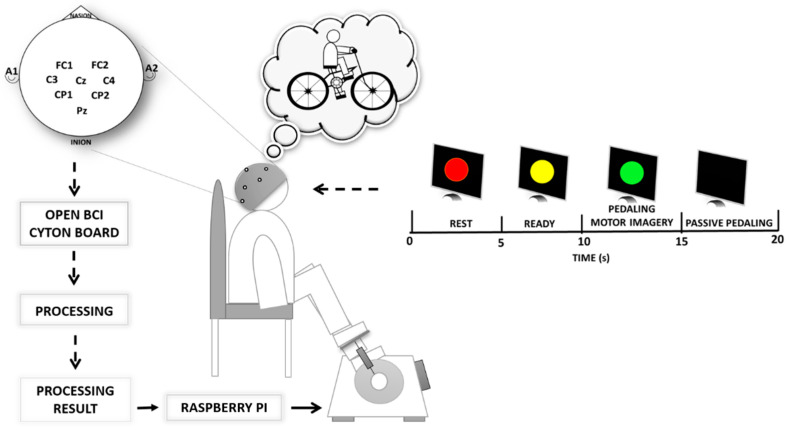
Experimental setup using our brain–computer interface scheme.

Our BCI (Figure 1) was built for the subject to turn on a stationary motorized pedal through movement imagination, receiving passive pedaling (movement mechanically realized by the pedal) as feedback for a period of 5 s.

Our BCI consisted of a low-cost wireless board (Open BCI Cyton, Copyright OpenBCI, New York, NY, USA), capturing 8 EEG channels with a 250 Hz sampling rate and using a notch filter at 60 Hz. This BCI was also composed of a notebook, a Raspberry Pi board, and a motorized pedal (Exerpeutic 7101 Activcycle Motorized Pedal Exerciser, Paradigm Health & Wellness, City of Industry, CA, USA), used as an output device.

The BCI operation was divided into two phases. The first phase was carried out to calibrate the BCI, where the subject was asked to perform two tasks: rest state and MI (without receiving any feedback—termed “MI” in open-loop conditions), each one for a period of 5 s. The second stage, termed the “online phase”, was carried out with the calibrated BCI to promote motor training, in which the subject performed MI to turn on the motorized pedal through the Raspberry Pi board, and consequently received feedback from the passive pedaling for a period of 5 s. As result, the participant felt a closed-loop performing MI and receiving feedback as a response. For this reason, we also used the term “MI” in closed-loop conditions in our study.

To guide the subject in each phase, instructions composed of four visual cues were displayed on the computer screen, indicating to them the instant to perform rest state (red cue) and motor imagery (green cue), as shown in Figure 1.

The design of this BCI was proposed based on the fact that EEG-based BCIs can be used for motor rehabilitation purposes through real or imagined movements of upper and lower limbs [39,40,41]. As a result, MI-based BCIs are an alternative to rehabilitate patients with severe motor deficits or no residual movements [40,42,43].

### 2.2. Data Recording and Signal Processing

The OpenBCI board was used to acquire the EEG signals from a 64-channel EEG cap of Ag/AgCl electrodes. There were 8 channels (FC1, FC2, C3, C4, Cz, CP1, CP2, and Pz) used in this study, which were located in accordance with the International 10–20 system (Figure 1). In addition, electrodes A1 Ground (GND) and A2 reference (REF) were placed on the left and right ears, respectively.

Initially, a calibration phase was carried out for the BCI learning, first collecting a training dataset formed by both the rest state and the MI in open-loop. This dataset was used by selecting epochs of 1 s from the raw EEG, taken as a reference 0.5 s after starting the rest state suggestion (red cue), and 0 s after beginning the suggestion for MI execution. Then, each epoch was processed by applying a band-pass filter with zero-phase, which was implemented through both the fast Fourier transform (FFT) and the inverse FFT (IFFT). Consequently, components outside the frequency range of 0.1 to 30 Hz were removed by multiplying a rectangular function into the frequency domain. Afterward, Riemannian geometry was used on covariance matrices (that were derived from the filtered epochs), to calculate the corresponding projection matrix onto the tangential space, useful for extracting spatial features. In sequence, the Pair-Wise Feature Proximity (PWFP) was applied to the feature set for dimensionality reduction in order to increase class discrimination and enhance the performance by applying Linear Discriminant Analysis (LDA) as a trained classifier. As a result, the calibrated BCI was used in the online phase to recognize the pedaling MI over periods of 1 s by overlapping 65 ms, providing an alternative route for participants to turn on the motorized pedal.

### 2.3. Protocol

There were 8 right-handed healthy subjects (7 males and 1 female, aged between 22 and 36 years) who participated in the experiments. Everyone had previous experience with pedaling, and 6 volunteers already had experience with BCI and biofeedback. The inclusion criteria consisted of having enough hearing, visual, cognitive, and language abilities to understand and follow the instructions.

**Figure 2:**
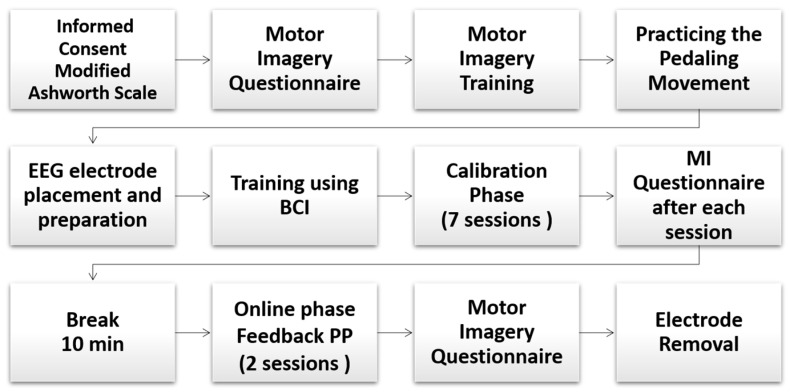
Sequence followed during the experimental protocol.

Figure 2 shows the sequence of the protocol. Firstly, we explained to each participant the main objective of our study and read the terms of informed consent. All participants gave their written informed consent in accordance with the Helsinki declaration. This research was approved by the Research Ethics Committee of the Federal University of Espırito Santo, Brazil (CAAE number: 64797816.7.0000.5542). Then, we administered a questionnaire about motor imagery, which was proposed by {Cho, 2017, EEG datasets for motor imagery brain–computer interface} [44].

**Figure 3:**
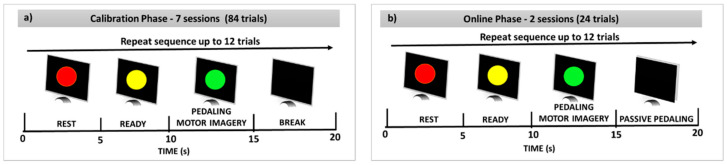
The experimental paradigm: (**a**) calibration phase, and (**b**) online phase.

Subsequently, the participant sat in a comfortable armchair in front of a 19-inch screen, placing their feet on the motorized pedal of the ergometer cycle. Before starting the experiment, a stage for motor imagination training was conducted, where each participant was asked to imagine riding a bicycle. At this stage, the participant further received passive movements using the pedal to feel the kinesthetic experience, to afterward imagine the same kinesthetic experience [44,45]. Kinesthetic motor imagery is described as the ability to imagine the execution of a movement by having an impression of muscle contraction and sensation during a real movement [46,47]. Simultaneously, the skin preparation and placement of EEG electrodes were done while the participant was instructed to continue training the kinesthetic motor imagery, following the visual cues (see Figure 1) provided by the BCI. The experiment sequence was divided into two phases: (1) the calibration phase and (2) the online phase. During the calibration phase, the raw EEG was collected from each participant, completing a total of 7 sessions, each one separated by a break interval of 3 min. Each session consisted of 12 trials, during which the participant was asked to perform two tasks per trial, such as rest state (red cue) and pedaling MI online (green cue), each one for a period of 5 s, following the sequence of visual cues shown in Figure 3a. During the red and yellow cues, the participant was asked to avoid voluntary movements, such as eyes and mouth movements. On the black screen, discrete mouth movements and eyes blinking were released. Therefore, the database for the BCI calibration was formed by a total of 84 trials.

After finishing each session, a questionnaire was filled out for the participant [44]. In the online phase, the calibrated BCI was used by each participant to turn on the motorized pedal by executing the pedaling MI and receive passive pedaling, forming a closed loop between the participant’s brain and pedal. This online phase consisted of 2 sessions, each one separated by a break interval of 3 min, and each session consisted of 12 trials, during which the participant was encouraged to turn on the pedal by MI throughout the green cue (see Figure 3b) in a period of 5 s, completing a total of 24 trials. The participant received similar instructions given in the calibration phase to avoid undesirable EEG artifacts. Additionally, the participant was instructed to not exert resistance on the pedal while receiving passive movements. Likewise, a questionnaire was filled out for each participant after finishing each session. Finally, we removed the electrodes.

### 2.4. Data Processing and Statistical Analysis

The cortical effect on participants using the BCI was studied through the analysis of significant event-related desynchronization (ERD) patterns into the time–frequency representation [5,48], relative power into the frequency domain, and EEG connectivity. Each method was implemented as follows.

#### 2.4.1. Significant ERD Patterns Analysis

The analysis of significant ERD patterns was carried out with trials in which each participant successfully turned on the pedal by MI and received passive movements as feedback. Then, we extracted segments of 7 s in length, aligning them at the instant that the motorized pedal was turned on (instant at 0 s). Each segment of 7 s contained the instant or epoch of 1 s recognized as MI (−1 a 0 s) to turn on the pedal, plus two other periods: (1) the period of 2 s (baseline) preceding the MI recognition and (2) the period of 4 s (passive movements) after the MI recognition. From these last periods, we only used the EEG data from −2.0 a −1.0 s as a reference or baseline, and from 0 to 3 s to represent the cortical activity produced by passive pedaling (PP). The segments of 7 s were first filtered in a frequency range from 0.1 to 40 Hz and analyzed, as done by [5,48], using the reference interval from −2.0 to −1.0 s to compute the relative power changes. Here, the significant ERD patterns were determined by applying the *t*- percentile bootstrap algorithm, considering a significance level between 0.01 and 0.20 (confidence intervals of 99% and 80%, respectively).

#### 2.4.2. Relative Power

In the frequency domain, we further compared the relative power changes between three conditions, such as the MI in the calibration phase, the MI in the online phase, and passive pedaling. First of all, we obtained a set of segments for each one of these conditions. In the sequence from the calibration phase, we extracted the first set of 84 EEG segments with up to 2 s in length (0 to 2 s), 0 s being the green cue start. Furthermore, all epochs of 1 s recognized as MI in the online phase that produced the motorized pedal movements were also used to form the second set, while the EEG data corresponding to the first 2 s of passive movements were selected to compose the third set. As a baseline, we used the period of 2 s preceding the MI tasks in both the calibration and online phases. Finally, each set of segments was processed by applying the FFT over periods of 1 s with an overlapping of 0.1 s for obtaining the average power spectrum.

Such as was done by [31], the FFT was applied to each set over periods of 1 s with an overlapping of 0.1 s for obtaining the average power spectrum after computing the relative power changes with respect to the baseline condition.

The Student’s *t*-test [49] was used on the relative power change to compare the cortical effect in closed-loop for both MI and passive movement conditions.

The Kolmogorov–Smirnov test for data normality verification was also used, which rejected the null hypothesis (*p*-value < 0.05) for the analyzed data, and thereby confirmed its normal distribution. Then, boxplots based on median values were obtained for statistical comparison among the rest state, the MI calibration phase, the MI online phase, and passive pedaling, considering a *p*-value of <0.05.

#### 2.4.3. Connectivity

The connectivity between the EEG locations for delta (0.1–4 Hz), theta (4–8 Hz), mu band (8–12 Hz), low beta (13–18 Hz), and high beta (19–30 Hz) bands was also studied here for three conditions: (1) pedaling MI in the calibration phase; (2) pedaling MI in the online phase; (3) receiving passive pedaling in closed-loop. A total of 84 trials with a duration of 5 s were analyzed for the first condition, using successive epochs of 1 s, every 0.5 s. The processing of all epochs was carried out as follows. First of all, the Common Average Reference (CAR) was applied to all EEG channels, followed by band-pass filtering to preserve the components of interest in each aforementioned frequency band. Then, the filtered epochs were transformed into the frequency domain by applying the FFT to compute the relative power band with respect to the full frequency range (0.1–30 Hz). Finally, Pearson’s correlation was applied to the pairwise EEG channels to analyze the connectivity between brain regions. Notice that for each condition, a set of epochs was obtained per frequency band, and therefore, a set of relative power bands was also achieved. To facilitate the connectivity representation, the square correlation matrix was calculated to find those EEG channels with strong connections (or the highest correlation), after computing the number of links to be preserved. These are defined as $N=\left(n^{2}-n\right)\times p_{Th}/2,\;$ where *n* is the total channels, and *p_Th_* is the threshold proportion (*p_Th_* = 0.05). Additionally, the strength of each selected channel was analyzed by calculating the accumulative correlation with respect to the other preserved channels. It is worth mentioning that the square correlation matrix was updated, setting zero values to those non-preserved pairwise EEG channels.

## 3. Results

### 3.1. Relative Power Analysis

**Figure 4:**
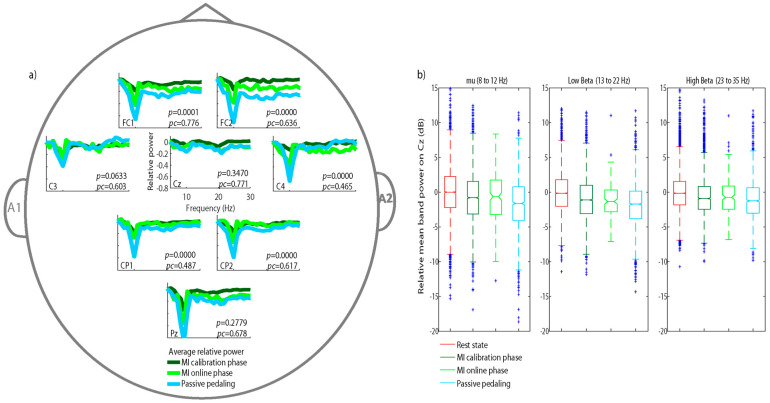
(**a**) Average relative power computed on 8 healthy subjects for three states, where *pc* and *p*-values compare the motor imagery (MI) calibration phase, online phase, and passive pedaling conditions in the online phase; (**b**) comparison of relative power over the Cz location analyzing the contribution of each frequency band when participants executed four tasks: rest state, MI during the calibration phase, instantly triggering the brain–computer interface (BCI) by MI, and receiving passive pedaling.

Our results demonstrate the feasibility of using EEG to identify brain electrical activity during MI tasks and passive pedaling while triggering the BCI. Figure 4b shows that for both the calibration and online phases, a significant power (*p* > 0.05) decreased over Cz during the conditions of MI and passive pedaling with respect to the rest state for the mu band, low beta, and high beta bands. However, the lower relative power over Cz (Figure 4a) for the mu band and low beta bands did not differ between passive pedaling and MI (*p* > 0.347). The highest power decrease was obtained for the low beta (around −0.25), when the participants received passive pedaling as feedback (Figure 4a). This power decrease for the beta band was also reported by Storzer et al. when studying active cycling. [31]. It is worth noting a peak at 10 Hz on the mu band in Figure 4a, which was more pronounced when the participant received feedback than when performing MI [31].

In addition, the results of the Student’s *t*-test revealed significant differences between passive pedaling and MI conditions on FC1, FC2, C4, CP1, and CP2 (*p* < 0.05) for the mu band (at 10 Hz). Figure 4 shows the most accentuated bilateral decrease for passive pedaling (PP).

### 3.2. ERD

**Figure 5:**
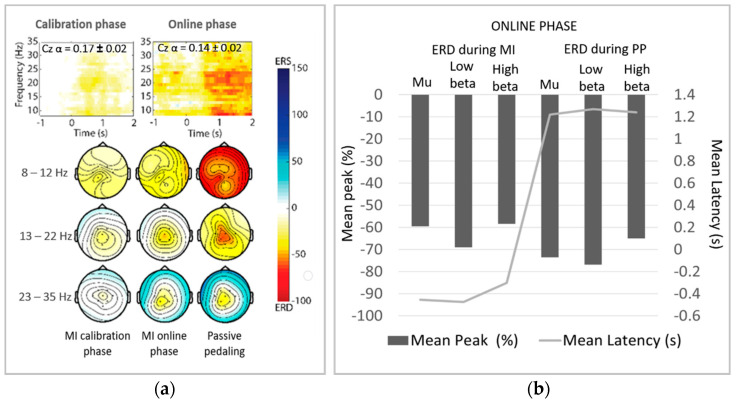
Significant event-related desynchronization (ERD) analysis. (**a**) Distribution of significant ERD changes in the time–frequency representation over Cz, and topographic maps of mean ERD power for the mu band, and the low and high beta bands, obtained when participants executed MI in the calibration phase and online phases. (**b**) Significant ERD power average for subject-specific bands and latency of ERD peaks during MI and passive pedaling in closed-loop.

The significant ERD patterns in the time–frequency representation (Figure 5a) were also analyzed. The average of the ERD peaks (see Figure 5b) on the mu band (8–12 Hz) and the beta (13–30 Hz) bands shows that ERD patterns were emphasized on Cz when the participants performed MI and received passive pedaling as feedback in closed-loop. The topographic ERD maps and ERD peaks also show the significant ERD focused around Cz for the low beta band. Similarly, significant desynchronization can be observed on the primary motor cortex when the participants received passive pedaling. It is worth commenting that cycling-related beta desynchronization has also been found in studies comparing walking versus cycling, and passive versus active cycling [30,31].

**Figure 6:**
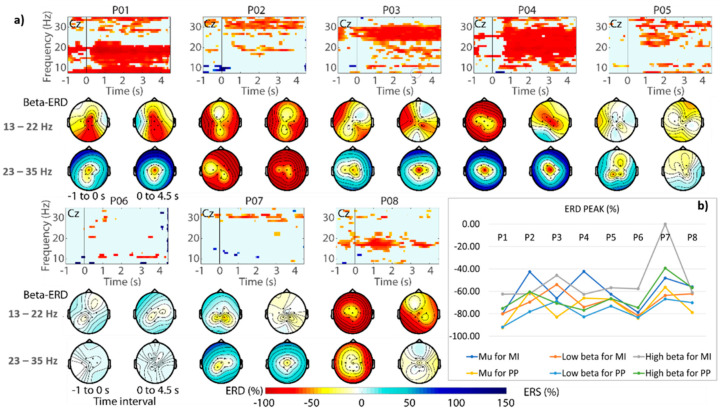
(**a**) Representation of significant ERD patterns using the time–frequency representation, where the intervals from −1.0 to 0 s and from 0 to 4.5 s are respectively related to MI before triggering the BCI and passive pedaling. (**b**) Average of ERD peaks for subject-specific frequency bands during MI and passive pedaling in closed-loop.

Figure 6a shows topographic ERD maps for all participants, which were obtained by calculating the average power of the low and high beta bands in the time–frequency representation. The significant ERD peaks during MI and passive pedaling are shown in Figure 6b.

For most participants, a power decrease over the primary motor cortex was obtained for the beta bands while performing MI and receiving feedback, as shown in Figure 5a,b. As a highlight, the highest significant ERD patterns focused on Cz (the foot area) were obtained for participants P01, P03, and P04. These findings agree with the reports from other researchers who studied foot movements by MI, also achieving cortical activity around Cz [5,8,30] Discrete significant ERD was obtained for participants P06 and P07, who also simultaneously generated ERD and event-related synchronization (ERS) when commanding the BCI and receiving feedback. These subjects presented ERD peaks in the mu band and the low beta band, not being identified as ERD peaks in the high beta band for participant P06 during MI conditions (Figure 6b).

### 3.3. Brain Connectivity

**Figure 7:**
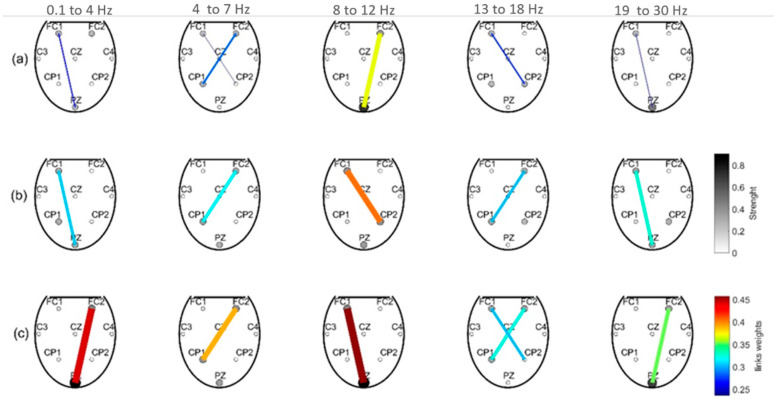
Connectivity between surface cortical areas considering flow and force between the electroencephalography (EEG) channels for the delta, theta, mu band, and beta bands, during three conditions: (**a**) MI calibration, (**b**) MI online, and (**c**) passive movements.

Our findings on brain connectivity considering the low beta band suggest a strong contralateral flow of information between the supplementary motor area (SMA) (FC1 and FC2) and the parietal central line (CP1 and CP2). For the high beta band, we observed a flow between FC1-Pz (MI in open-loop) and FC2-Pz (passive pedaling), shown in Figure 7. For the mu band, the SMA connected greatly with Pz when the participants performed MI in open-loop and received PP in closed-loop. In addition, a strong flow between FC1 and CP2 was evidenced when these participants executed MI in closed-loop. Notice that this band presented the strongest flow for the three conditions studied. These achievements agree with other studies analyzing brain connectivity during movement and feet MI [33,34].

Similar connectivity for the low beta band was observed for the theta band (4–7 Hz). This is an interesting finding since initial EEG studies showed that cortical activation on the theta band is elicited in people solving problems, learning, and during perceptual processing [50].

Lastly, our BCI has a latency of 328 ms to translate a neural state (MI tasks) into a command to turn on a motorized pedal, which provides feedback by passive movements for a period of 5 s at 60 rpm. In order to obtain better results, we instructed our participants to use kinesthetic sensation when imagining movement, with passive movement offered as feedback. The mean accuracy (ACC) of our BCI was calculated as 68.86 ± 1.23% and the Kappa was 0.38 ± 0.02 in the calibration phase, whereas for the online phase, the ACC was 91.67 ± 5.51% (for more information on this topic, see reference Romero-Laiseca et al.) [51].

## 4. Discussion

This work studied the role of a few EEG locations over the primary motor cortex when volunteers used our BCI to turn on a motorized pedal by MI and receive passive movements. We noted a strong desynchronization and more acute ERD peaks for most participants, as well as frontoparietal connectivity with emphasis on the beta bands. The achieved results highlight the importance of low and high beta bands for pedaling MI and passive pedaling movement, in accordance with other studies that also associated these bands with pedaling exercises. These findings are interesting due to the fact that mu and beta rhythms are functionally related to the main sensorimotor systems [50], which are mainly activated during motor preparation and execution [52].

Regarding cycling, the cortical involvement of motor control was previously studied by the EEG technique during cycling, where it was observed that active movements, as opposed to passive movements, led to a stronger power decrease on the beta band over the sensorimotor cortex [30]. Comparing walking versus cycling, researchers found, over Cz, the highest power decrease on the beta band (13 to 15 Hz) in cycling conditions, while for walking, the highest decrease was obtained on the mu band (8 to 12 Hz) over the primary motor cortex [31]. Other studies have also devoted themselves to studying the cycling effect on brain activity, finding cortical activation on the primary motor cortex [29,32]. These authors proposed a BCI that decodes brain activities to control a gait training exoskeleton, obtaining features to discriminate MI and rest state, with a higher contribution from the primary motor cortex, especially on Cz [4], whereas a negative peak on the relative power was found bilaterally for the mu band (see Figure 4). This result agrees with the reports provided by another study using Positron Emission Tomography (PET), where the authors observed bilateral activations on the primary sensory cortex, the primary motor cortex, and the supplementary motor cortex during active and passive pedaling. Similarly, other research bilaterally found activation on the supplementary motor cortex when volunteers performed MI [32]. On the other hand, the highest negative peak on the relative power was achieved when participants received feedback through passive pedaling in closed-loop, in response to answering their MI. This cortical effect suggests for us a higher sensory input commanding our BCI (see Figure 4a). In this way, a study comparing active and passive cycling found that most cortical activation during passive cycling is elicited by sensory feedback because of the moving limbs [30]. Another study carried out with a robot for active/passive gait assistance showed that the mu rhythm is suppressed on the central midline areas during the active gait compared to passive walking, suggesting that this effect probably occurred due to the increased sensory feedback from muscles [53].

Our brain connectivity analysis revealed an exchange of information between SMA and the parietal lobe, with contralateral flows for both MI and passive pedaling tasks. Likewise, Athanasiou et al. (2012) [34] analyzed brain connectivity, finding stronger activation on SMA during foot MI, and the information flow was elicited contralaterally from SMA towards the foot area on the primary motor area [34]. This finding agrees with the literature supporting the regulatory role of SMA during motor planning [35,50,52]. We also noted that the FC1, FC2, CP1, and CP2 locations presented the highest strengths for the low beta band. For this later band, the highest power decrease and the most accentuated ERD peaks were obtained. This achievement correlates with a previous study aiming to distinguish leg flexion and extension, where the selected EEG channels were FC5 and CP6, located on the Brodmann areas (40 and 44), which are specialized for motor planning function and somatosensory integration [4].

Considering the Hebbian learning principle [54], the maximum feedback delay of our BCI is acceptable, with a latency of 328 ms. It is worth mentioning that the feedback delay is considered a fundamental characteristic to induce changes for enhancing brain plasticity by means of BCIs, where the participants should feel a real closed loop. [54,55]. Additionally, our BCI responded successfully to the participants’ intentions with a mean ACC of 91.67% during the online phase, which is desired since good accuracy may result in smaller feedback delays [53].

In this preliminary study on the behavior of cortical excitability and brain connectivity in which participants controlled a BCI based on motor imagery tasks and pedaling exercise, we believe that our findings may contribute to this area in future research. As a limitation of this work, only 8 individuals were analyzed. Thus, the number of participants should be increased in future works.

## 5. Conclusions

Topography maps, relative power decreases, and ERD peaks showed cortical activation on the primary motor cortex for most participants during pedaling motor imagery and passive pedaling, while we observed contralateral brain connectivity between SMA and the parietal lobe, with emphasis on the low beta band. We believe that this preliminary study brings interesting results that may contribute to future research to improve the effectiveness of BCI technologies based on cycling exercises.

## Data Availability

Not applicable.
